# A genome-wide association study of germline variation and melanoma prognosis

**DOI:** 10.3389/fonc.2022.1050741

**Published:** 2023-01-19

**Authors:** Vylyny Chat, Sasha Dagayev, Una Moran, Matija Snuderl, Jeffrey Weber, Robert Ferguson, Iman Osman, Tomas Kirchhoff

**Affiliations:** ^1^ Perlmutter Cancer Center, New York University School of Medicine, New York, NY, United States; ^2^ Department of Population Health and Environmental Medicine, New York University School of Medicine, New York, NY, United States; ^3^ The Interdisciplinary Melanoma Cooperative Group, New York University School of Medicine, New York, NY, United States; ^4^ Department of Pathology, New York University School of Medicine, New York, NY, United States; ^5^ Ronald O. Perelman Department of Dermatology, NYU Grossman School of Medicine, New York, NY, United States

**Keywords:** cutaneous melanoma, genome-wide association study, single nucleotid polymorphisms, cancer survival, prognostic biomarkers

## Abstract

**Background:**

The high mortality of cutaneous melanoma (CM) is partly due to unpredictable patterns of disease progression in patients with early-stage lesions. The reliable prediction of advanced disease risk from early-stage CM, is an urgent clinical need, especially given the recent expansion of immune checkpoint inhibitor therapy to the adjuvant setting. In our study, we comprehensively investigated the role of germline variants as CM prognostic markers.

**Methods:**

We performed a genome-wide association analysis in two independent cohorts of N=551 (discovery), and N=550 (validation) early-stage immunotherapy-naïve melanoma patients. A multivariable Cox proportional hazard regression model was used to identify associations with overall survival in the discovery group, followed by a validation analysis. Transcriptomic profiling and survival analysis were used to elucidate the biological relevance of candidate genes associated with CM progression.

**Results:**

We found two independent associations of germline variants with melanoma prognosis. The alternate alleles of these two SNPs were both associated with an increased risk of death [rs60970102 in MELK: HR=3.14 (2.05–4.81), p=1.48×10^-7^; and rs77480547 in SH3BP4: HR=3.02 (2.02–4.52), p=7.58×10^-8^, both in the pooled cohort]. The addition of the combined risk alleles (CRA) of the identified variants into the prognostic model improved the predictive power, as opposed to a model of clinical covariates alone.

**Conclusions:**

Our study provides suggestive evidence of novel melanoma germline prognostic markers, implicating two candidate genes: an oncogene MELK and a tumor suppressor SH3BP4, both previously suggested to affect CM progression. Pending further validation, these findings suggest that the genetic factors may improve the prognostic stratification of high-risk early-stage CM patients, and propose putative biological insights for potential therapeutic investigation of these targets to prevent aggressive outcome from early-stage melanoma.

## Introduction

Cutaneous melanoma (CM) is the most lethal form of skin cancer with a steadily increasing incidence in the United States (1, 2). It is estimated that 106,110 new CM cases will be diagnosed in 2021, and 7,180 of those will die of the disease (2). Surgical excision of the primary tumor in early stages commonly serves as a curative strategy. However, a significant fraction of tumors unexpectedly recur at more advanced stages, thus contributing to CM being the most lethal form of skin cancer. The American-Joint Committee on Cancer (AJCC) staging scheme is commonly used to guide treatment and follow-up prognostication; yet AJCC staging does not explain the significant variability in long-term CM survival amongst melanoma patients diagnosed at early stages (3, 4). This points to a need for a complementary identification of more personalized CM prognostic markers to improve disease surveillance, select patients for neoadjuvant or adjuvant treatment strategies, and provide patient stratification for clinical trials.

While the current AJCC 8^th^ staging system includes a comprehensive list of relevant clinical factors (eg. Breslow tumor thickness, ulceration, and positivity of sentinel lymph nodes) that impact melanoma progression, it does not account for baseline molecular features. In this regard, the role of germline genetics in tumor progression has been suggested by the intriguing evidence of similar survival patterns in cancer patients from the same families (5, 6). Several reports have shown that specific host germline genetic variants modulate melanoma outcomes. This includes variants of melanoma risk (7–9), DNA repair (10), and results from a seminal study focused on immunomodulatory gene variation as a surrogate of melanoma survival (11). Some of these candidates hold promise for clinical applicability, as independent prognostic markers (12). While highly promising, the evidence in support of germline prognostic markers reported in these and other (13–15) studies, stems from the candidate gene approach focused on a handful of plausible pathways with limited scope. This approach does not capture the complex biological nature of CM etiology and progression. While genome-wide scans of melanoma risk have been previously conducted and revealed a number of important risk variants (16–19), GWAS studies focused on prognostic markers (survival/progression/recurrence) have been sparse, mainly due to a lack of sufficient statistical power of cohorts with comprehensive longitudinal follow-up data. In this study, we conducted, a genome wide association study (GWAS) to comprehensively elucidate the role of common germline variants in melanoma clinical outcomes using two independent cohorts of immunotherapy-naive CM patients with extensive clinical long term follow-up information, treated at New York University Langone Health (NYULH).

## Methods

### Study population

We initially employed a cohort of 551 immunotherapy-naive melanoma patients for the discovery analysis who were prospectively enrolled in the Interdisciplinary Melanoma Cooperative Group (IMCG) at New York University Langone Health (NYULH) from 2002-2018. The IMCG clinicopathological, follow-up information, and biospecimen collection protocols are described elsewhere (20). In brief, for each patient, blood samples along with relevant clinical and demographic information such as age at diagnosis, sex, AJCC tumor staging, tumor anatomic site, and self-reported ethnicity were collected at the time of enrollment. Patients’ follow-up information was collected every 6 months during clinic visits. In this study, in order to reduce heterogeneity in patient characteristics, disease subtypes, and treatments that may preferentially modulate melanoma outcomes, we included only Caucasian patients (~98% in our cohort) diagnosed with less advanced (stage I-III) CM and with no history of immunotherapy. Acral melanomas were excluded, due to different prognosis and etiology, compared to other CM subtypes (21–23). All patients signed informed consent prior to study initiation, and the Institutional Review Board (IRB) at NYU approved the study.

To validate the findings from the discovery stage, we ascertained a subsequent independent cohort of 550 CM patients, from the same IMCG population (2002-2018), using protocols as described in the discovery subset. To maintain the reproducibility, again, for the validation cohort, we have selected only immunotherapy-naïve patients, with less advanced stages (I-III), of white European ancestry, and excluding other etiologies (e.g. acral melanomas), as described for the GWAS discovery subset. We noted that the AJCC 8^th^ staging distribution (**Table 1**) was balanced between the two sets. The overall melanoma-related death rate was 6.2% (**Table 1**) which is lower than expected. Given that patients who need immunotherapy would have increased probability of death, the exclusion of such patients from this study would likely explain lower-than-expected death rate observed among our patient population.

**Table 1:** The clinical and demographic information for the patient population in the discovery, validation and pooled cohort .

Characteristics	Pooled (N=1,101)	Discovery (N=551)	Validation (N=550)	p-value*
Age at diagnosis (median; range)	60 (15–97)	59 (15–97)	61 (18–96)	0.08
Follow-up years (median; range)	5.8 (0.1–49.6)	5.6 (0.3–34.8)	5.8 (0.1–49.6)	0.2
Sex N (%)				
* Male*	611 (55.5)	307 (55.7)	304 (55.3)	0.9
AJCC 8^th^ stage N (%)				
* I*	797 (72.4)	393 (71.3)	404 (73.5)	0.4
* II*	178 (16.2)	88 (16.0)	90 (16.4)	
* III*	126 (11.4)	70 (12.7)	56 (10.2)	
Primary tumor anatomic sites N (%)				
* Axial*	612 (55.6)	306 (55.5)	306(55.6)	
* Extremity*	477 (43.3)	237 (43.0)	240 (43.6)	1
* Unknown*	12 (1.1)	8 (1.5)	4 (0.7)	
Number of events N (%)				
* Overall mortality*	122 (11.1)	65 (11.8)	57 (10.4)	0.5
* Melanoma-Death*	68 (6.2)	35 (6.4)	33 (6.0)	0.9

* p-values were estimated from Wilcoxon rank sum and chi-square tests for non-parametric continuous and categorical variables, respectively.

### Sample processing, genotyping, and quality control

The genomic DNA was isolated from whole blood samples with Qiagen DNeasy 96 blood & tissue kit. To quantify DNA concentration and to assess DNA integrity, Qubit and gel electrophoresis were used. There was no evidence of DNA degradation and all the samples included in the study had a minimum concentration of 10ng/µl. For GWAS genotyping, Illumina Global Screening Array Multi-disease (GSA-MD V3.0) was used. Prior to genotyping, the samples were tested using a panel of 25 previously curated variants (24) for the purpose of identity sample tracking (ID panel). The ID panel genotyping was performed by the Sequenom MassArray System (Agena Bioscience Inc, CA, USA) as per the manufacturer’s protocols. High concordance (>99%) between ID panel and GSA-MD V3.0 was observed, minimizing the possibility of sample mismatches.

We used Genome Studio V2.0 to convert raw intensity signals of 730,059 genomic variants on the GSA-MD V3.0 array into hard call genotypes with a GenCall threshold of 0.15. Based on the control dashboard of Genome Studio, no evidence of cross-contamination nor issues with internal control probes were detected. PLINK Input Report Plug-in v2.1.4 was used to export genotype calls to PLINK format for downstream analyses.

As part of quality control (QC), with PLINK 1.9/2.0 (25), we computed principal component scores (PC scores detailed in (26)), and excluded study participants of non-European ancestry (> ± 3SD from the mean PC scores). We also removed samples with reported vs. imputed sex discrepancy, or with enriched rate of heterozygosity (>3SD from the mean) as well as samples of cryptic relatedness (Pi-hat >0.25). For SNP filtering, we removed non-autosomal variants, and variants with missing rate > 5%, minor allele frequency <5% and Hardy-Weinberg equilibrium (HWE) <1×10^-7^. We further removed samples with poor call rate (<95%). After filtering, we imputed the filtered GSA-MD variants using the open-source Michigan imputation server with the haplotype reference consortium (HRC v1.0) as a reference panel (27). We performed post-imputation QC including filtering out variants with imputation score R^2^ ≤ 0.5, MAF <5%, missing rate > 90% or HWE <10^-7^were further filtered out. After the quality control steps, the number of patients remaining in the final analyses for the discovery and validation cohort were 522 and 520, respectively (N pooled = 1,042). All QC criteria were applied as per standard GWAS pipelines (28, 29).

### Statistical analysis

We assessed the heterogeneity of patient characteristics using Wilcoxon-rank sum and chi-square tests for continuous non-parametric variables and categorical variables, respectively. A multivariable Cox-proportional hazard regression model was used to test the associations of approximately 5 million post-QC imputed germline variants with melanoma outcomes adjusted by relevant demographic and clinico-pathological variables such as age at diagnosis, sex, tumor anatomic site, tumor stage, and top 3 principal components (PCs). The effect estimates were reported from an additive genetic model as a hazard ratio (HR) with 95% confidence interval (95%CI) and *Wald test* p-values. The main outcome of interest was overall survival (OS), defined as time from melanoma diagnosis to death or last follow-up. As a sensitivity analysis, we also explored the effect of the top-ranked variants with melanoma-specific survival (CMSS). All survival analyses were performed with an R package *gwasurvivr 1.10.0* (30) using PLINK-format genotypes as inputs.

In the discovery stage, we selected OS-associated variants with nominal p-value < 10^-4^, and Bayesian false discovery probability (BFDP) <0.1. We used a suggestive p-value of significance at the discovery stage (rather than GWAS level of significance p<5×10^-8^) to expand the number of variants to be included in the validation stage 2. BFDP was used instead of the *Bonferroni* multiple testing adjustment because the tested markers were imputed with expected high-linkage disequilibrium and hence, were not independent. We computed BFDP using the R package *gap* (*genetic analysis package*), assuming prior probability 0.1 and estimated effect size HR ~ 3, as suggested by prior studies of cancer prognostic germline biomarkers (31–33). Based on these threshold criteria, 1,377 variants were selected for validation. In the pooled analyses we focused on variants with p<0.05 in the validation and p<5×10^-7^, as a suggestive genome-wide threshold of significance, proposed previously in cancer survival GWAS studies (34), which are usually limited by analytical power. Complete results of all replicated variants in the pooled analysis are shown in **Supplementary Table S1**. We further estimated the time-dependent AUC of the Cox proportional regression models using the *timeROC* R package (35) to quantify the predictive utility of the OS germline markers. Stratified analyses to assess heterogeneity in the effect of the identified OS-associated germline variants were conducted on a multiplicative scale by generating cross-product terms of interaction between genotypes and clinical covariates using R.3.6.3 (36).

### Transcriptomic profiling of candidate gene markers in melanoma progression

To further elucidate the biological relevance of the identified candidate genes in melanoma progression, we utilized tumor transcriptomic data available from 46 CM patients treated at NYULH (16 of which overlapped with the patient population in this study). RNA was prepared from the formalin-fixed paraffin-embedded (FFPE) tissue slides of N=26 primary and N=20 metastatic tumor samples, using RNAeasy FFPE kit (Qiagen). RNA sequencing was performed with a 150 bp paired-end configuration to obtain ~30 million sequencing reads per sample. The raw fastq files were hard-trimmed with cutadapt (37) (minimum of 50bp and maximum of 80bp) and mapped to the reference genome with STAR aligner (38). RSEM (39) and DESeq2 normalization method (40) were used to quantify and normalize gene expression values, respectively. The normalized gene expression of the candidate genes were log2-transformed, and compared between primary vs. metastatic tissues using a Wilcoxon-rank sum test.

## Results

### Characteristics of the study cohort

Demographic and clinicopathological characteristics of both the discovery and validation cohorts were comparable (**Table 1**). Patients in the discovery cohort were slightly younger than those in the validation cohort (median age 59 vs. 61 respectively). Median follow-up time in the pooled data was 5.8 years (range 0.1–49.6). The majority of the study participants were male (55.5%) with AJCC 8^th^ stage I (72.4%) and axial primary tumor site (55.6%). The percentage of overall mortality and melanoma-related death were 11.1% and 6.2%, respectively.

### Genome-wide association study of genetic variation associated with melanoma prognosis

We tested the association of germline variants with overall survival (OS) using multivariable Cox proportional hazard model adjusting for age at diagnosis, sex, AJCC stages, tumor anatomic sites, and top 3 PC scores. From the discovery cohort, we identified 1,377 variants associated with overall survival at p < 10^-4^, and BFDP <0.1. Among the identified signals in the discovery stage, 46 loci were identified in the validation stage (p<0.05, with the same directionality of effect estimate HR that was observed in the discovery cohort). We performed SNP clumping of these loci (Linkage disequilibrium LD R^2^ threshold = 0.6), and retained 13 independent index variants (**Table S1** complete results in **Table S2** available here: https://figshare.com/s/860465237a7250ab82f8. Among the 13 validated variants, two markers had a p-value in the pooled analysis surpassing p < 5×10^-7^ (**Table 2**). The most significant association was observed for rs60970102: a minor T allele was associated with significantly worse survival (HR=3.14; 95%CI: 2.05–4.81; p=1.48×10^-7^). The second most significant association was found for rs77480547: an alternate allele A of rs77480547 increased risk of death by 3.02 times (95% CI: 2.02–4.52; p=7.58×10^-8^). Manhattan plot of the pooled analysis is shown in **Figure S1**. Kaplan-Meier survival curves according to the two significant variants are shown in **Figure S2**.

**Table 2:** The two most significant associations of germline variants with overall survival (OS) and cutaneous-melanoma specific survival (CMSS) identified in a GWAS in the discovery, validation and pooled cohorts.

Top SNPs	Chr : Pos:Ref/Alt	MAF	Discovery		Validation		Pooled	
			HR (95% CI)	P-value	HR (95% CI)	P-value	HR (95% CI)	P-value
**rs60970102**								
*OS*	9:36708133:C/T	0.07	3.83 (2.20–6.65)	1.94×10^-6^	2.47 (1.26–4.83)	0.008	3.14 (2.05–4.81)	**1.48×10^-7^**
*CMSS*			4.14 (1.90–9.04)	0.0003	2.56 (1.15–5.52)	0.02	3.30 (1.91–5.70)	1.92×10^-5^
**rs77480547**								
*OS*	2:235831702:G/A	0.06	3.41 (1.88–6.19)	5.48×10^-5^	2.48 (1.23–5.01)	0.01	3.02 (2.02–4.52)	**7.58×10^-8^**
*CMSS*			5.50 (2.49–12.1)	2.47×10^-5^	3.22 (1.26–8.21)	0.01	4.20 (2.50–7.04)	**5.60×10^-8^**

Cox-proportional hazard regression models assuming an additive genetic effect were adjusted for age at diagnosis, sex, AJCC 8^th^ stages, tumor anatomic sites and top 3 PCs. Hazards ratio (HR ± 95% CI) and wald-test p-values are reported. HR > 1 indicated increased risk of death associated with an alternate allele. Bolded p-values referred to statistically significant associations at p <5×10^-7^.

To test if the identified variants are independent markers of melanoma OS, we added the two variants into a multivariable Cox proportional hazard model adjusting for other covariates including age at diagnosis, sex, tumor anatomic sites, AJCC stages, and top 3 PCs. We found that both markers (rs60970102 and rs77480547) remained statistically significant at p<5×10^-7^ (p= 7.71×10^-7^ and 2.72×10^-7^, respectively), suggesting the associations were independent of one another, and of other clinical covariates (**Table S3**).

We also investigated if these two OS markers were predictive of CM survival (CMSS), defined as time from diagnosis to death due to melanoma. Using multivariable Cox-proportional hazard model adjusting for the same covariates as the OS model in the pooled cohort, we observed an enhanced signal for rs77480547 (HR=4.20; 95%CI: 2.50–7.04; p=5.60×10^-8^). For rs60970102, the association effect size remained comparable, albeit slightly less statistically significant (HR=3.30; 95%CI: 1.91–5.70; p=1.92×10^-5^) (**Table 2**).

### The effect of combined risk alleles on melanoma prognosis

Next, we assessed the combined effect of these two variants by adding the number of risk alleles (the minor alleles) assuming an additive genetic model to generate combined risk alleles (CRA) for each patient. The CRA in our cohort ranged from 0–3, representing the absence of any risk allele for the two markers (zero), or the number of risk alleles from either marker (1–3) (**Table 3**). A multivariable Cox proportional hazard model adjusting for other covariates found an increment by one of the number of risk alleles to be associated with 2.99 times increased risk of death (95%CI: 2.24–3.98; p =9.21×10^-14^).

**Table 3:** The combined effect of risk alleles of 2 most significant associations (rs60970102 and rs77480547) with CM OS detected in the GWAS study, assuming an additive genetic model and categorizing by risk groups.

Combined risk alleles (CRA)	N (%)	Additive genetic model		Stratified by risk groups			
		HR (95% CI)	P-value^*^	Risk Groups	N deaths (%)	HR (95% CI)	P-value^*^
0	711 (76.3)	2.99 (2.24–3.98)	9.21×10^-14^	Low	58 (8.2)	Ref.	Ref.
1	196 (21.0)			Medium	36 (18.4)	3.64 (2.33–5.69)	1.43×10^-8^
2	23 (2.5)			High	9 (36.0)	8.30 (3.99-17.27)	1.48×10^-9^
3	2 (0.2)						
						**p-trend**	2.18×10^-13^

^*^ Models adjusted for age at diagnosis, sex, tumor stage, tumor anatomic sites, and top 3 PCs. In the additive genetic model, the composite germline risk alleles (CRA) was treated as a continuous variable. We further categorized patients based on their CRA scores (low risk CRA=0, medium risk CRA=1, and high risk CRA >1), and tested for the association of the combined effect on OS with low risk group as reference. Individuals with the highest number of CRA has the highest risk of death as reflected by both percentage of death and HR in the high risk group compared to medium and low risk. P-trend: p-value of trending significance assuming the three risk groups as continuous variables.

**Figure 1:**
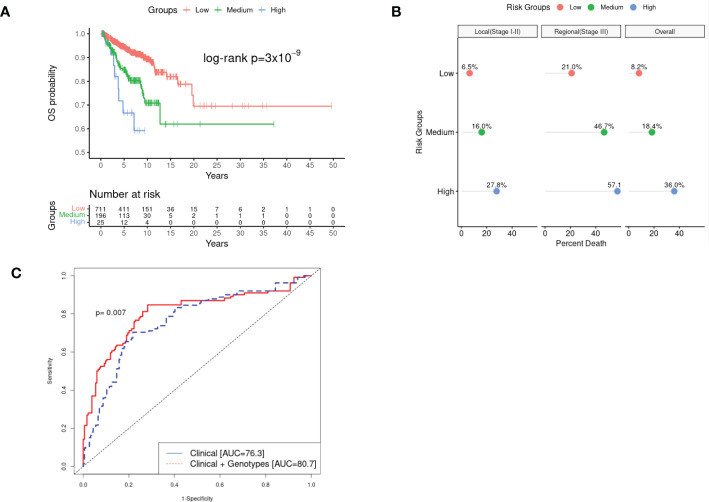
Kaplan-Meir survival (KM) curves, percentage of overall mortality, and estimated Area under the Curve (AUC) of the combined effect of rs77480547 and rs60970102 on CM OS detected in the GWAS study. Panel **(A)** plotted the survival curves of patients stratified by low risk (CRA=0), medium risk (CRA=1) and high risk (CRA>1) groups. We noted a clear dosage pattern of CRA effect on OS, further illustrated in Panel **(B)** by the linear increase in proportion of death as the CRA increased, for both local (stage I-II) and regional (stage III) disease over the median 5.8 year follow-up time. Panel **(C)** showed a statistically significant improvement in the estimated 10-year AUC (pooled cohort) as we incorporated CRA into the predictive model compared to the model of demographic and clinical variables alone (p=0.007).

We also explored the combined effect of these two markers by grouping patients into three risk groups [low risk (CRA=0) as reference, medium risk (CRA=1), and high risk (CRA >1)]. Patients in the medium CRA group were at a 3.64 increased risk of death compared to the reference (95%CI: 2.33–5.69; p=1.43×10^-8^), and the risk effect was even more pronounced in the high-risk group (HR= 8.30; 95%CI: 3.99–17.27; p=1.48×10^-9^), revealing a combinatorial effect for the two putative markers combined (**Figures 1A–B** **)**.

We estimated the time-dependent area under the curve (AUC) in the pooled cohort, and compared the Cox-proportional hazard model with only demographic/clinical information (age at diagnosis, sex, AJCC 8^th^ stages, tumor anatomic sites, and top 3 PCs); “model 1” vs. model 1 + genotypes (“model 2”). The addition of the genotype information into the predictive model significantly improved the 10-year AUC from AUC= 76.3 in model 1 to AUC=80.7 in model 2 (p=0.007) (**Figure 1C**). We estimated 10-year AUC, rather than 5-year AUC because most of our study subjects were early-stage patients with low mortality in the early years. Based on the ANOVA test of model fit, we also showed that adding genotype information to model 2 significantly improved the overall fit of the data (p=2.40×10^-10^) (**Table S4**).

We further performed stratified analyses to assess if the effect of the CRA on melanoma OS was modified by relevant clinical covariates such as age at diagnosis (≥ median 60 vs. <60), sex (Female vs. male), AJCC stage (local disease [stage I-II] vs. regional [stage III]) and tumor anatomic sites (axial vs. extremities) (**Figure S3**). Each stratified analysis consistently showed comparable effect estimates for the association of CRA and risk of death, and there was no statistically significant interaction between CRA and any of the tested clinical covariates.

### The gene-based analyses, functional annotation, and biological relevance

We used the Haploreg v4 database (41) to show that both putative prognostic markers of OS (rs60970102 and rs77480547) map to the intergenic regions in the vicinity of MELK (30KB 3’ of MELK) and SH3PB4 (29KB 5’ of SH3BP4), respectively (**Table S1**, **Figure S4**). To further explore the relevance of the two genes as melanoma prognostic candidates, we performed a gene-based multi-marker analysis of genomic annotation (MAGMA) (42). There were comparable numbers of variants mapped (110 KB upstream and 40 KB downstream window) to MELK (~280 SNPs) and SH3BP4 (~480 SNPs) in the discovery, validation, and pooled analysis (**Table S5**). Interestingly, by integrating multi-marker signals into a combined gene-based effect, we also observed a statistically significant association with OS for the two candidate genes in the discovery cohort (MELK p=1.75×10^-4^ and SH3BP4 p=0.007) with reproducible associations in the validation phase (p= 0.02 and p=0.009, respectively). In the pooled analysis, the statistical significance of the association of signals from these two genes with survival was further increased (MELK p-value =1.26×10^-5^, and SH3BP4 p=1.13×10^-5^), and strongly driven by the top 2 candidate variants (rs60970102 and rs77480547). We also queried the ENCODE database (43) to assess if these two candidate SNP markers were mapped within important regulatory regions. We found that rs77480547 mapped within close proximity (~1KB) to the DNase hypersensitivity cluster and various histone marks (H3K4me3 and H3K27ac). While there was no evidence of cis-regulatory elements (cCREs) for rs60970102 from ENCODE, according to the cancer sQTL (splicing-quantitative trait loci) database (44), rs60970102 was reported to preferentially modulate somatic MELK isoforms in colon cancer. This suggests that rs60970102 may impact alternative splicing patterns of MELK, providing a putative biological mechanism by which the observed genetic association in MELK may affect melanoma progression.

**Figure 2:**
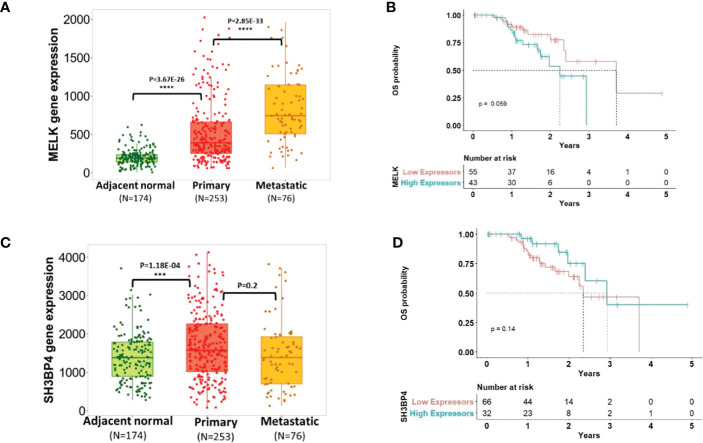
Transcriptomic profiling of the candidate gene expressions and melanoma progression. Panel **(A+C)** showed the expression of MELK and SH3BP4 comparing adjacent normal vs. primary vs. metastatic tumor tissues [adapted from tnmplot.com (45)]. Panel **(B+D)** plotted Kaplan-Meir survival curves [adapted from the Human Protein Atlas (46)] of early-stage CM patients with low vs. high expression of MELK and SH3BP4 in primary tumors. High expression of MELK was associated with worse OS (log-rank p=0.059), while the inverse trend was observed for SH3BP4 (albeit not statistically significant p=0.14), reflecting their roles as an oncogene and a tumor suppressor, respectively.

To investigate the biological relevance of the candidate genes (MELK and SH3BP4) in CM progression, we queried TNMplot (45), leveraging gene array data from 3180 expression datasets from the Gene Expression Omnibus of the National Center for Biotechnology Information (NCBI-GEO). This tool compares differential gene expression in adjacent normal vs. primary and metastatic tumor tissues for several cancer types (45). We found that in skin cancer, both MELK and SH3BP4 were significantly overexpressed in primary tumor compared to adjacent normal, suggesting its role in tumor initiation (**Figures 2A, C**). While there was no evidence of differential SH3BP4 expression in metastatic compared to primary tumors (p=0.20), upregulation of MELK was observed in metastatic tissues (p=2.85×10^-33^), providing further support that MELK may stimulate tumor progression. To test if the expression of these genes was directly associated with patient overall survival (OS), we queried the Human Protein Atlas (46), in which the TCGA early-stage CM patients’ clinical and gene expression data were used. Despite the small sample size of N=98 stage I-III TCGA samples with available primary tumor gene expression, we noted a trend of the association of high MELK expression [dichotomized using the optimal expression threshold to best separate the KM curves from the Human Protein Atlas (46)] with less favorable CM survival (median OS=2.25 years in MELK high expressors vs OS=3.71 years in MELK low expressors; log-rank p=0.059; **Figure 2B**). Interestingly, the inverse association pattern was observed for SH3BP4, albeit not statistically significant (log-rank p=0.14; **Figure 2D**).

## Discussion

In this study, we conducted GWAS to explore host germline variants as biomarkers of melanoma prognosis. Capitalizing on clinic-based long-term follow-up data of early-stage immunotherapy-naïve CM patients (not treated by immune-checkpoint inhibitors) with extensive clinical annotation and follow-up information, we discovered two variants reproducibly associated with CM OS in both the discovery and validation phases with the most significant association(p <5×10^-7^) in the pooled analysis.

Recent candidate gene studies, all derived from the MD Anderson Cancer Center study (MDACC) and the Nurse Health Study (NHS)/Health Professionals Follow-up Study (HPFS) cohorts, identified common germline markers of melanoma progression in several important biological pathways, including peroxisome, glycosylation, and folate metabolism (13–15). These studies reported novel candidate prognostic CM biomarkers that were validated in an independent study cohort. In-silico functional annotations of these findings further indicated the plausible biological relevance providing convincing evidence of the role of these germline variants in the modulation of CM-related gene expression. These candidate variants, however, were not replicated in our study (**Table S6**), which may be due to several factors. One possible explanation may be the difference in tumor staging assessment in the MDACC cohort or a lack of staging information in the NHS/HPFS cohort. Another contributing factor may be the high proportion of advanced-stage patients in these prior studies (17% with distant metastasis in the MDACC cohort) (14), or the limited follow-up information, which may each contribute to the differences in validating the associations in these studies. In contrast, our study focused on less-advanced immunotherapy-naïve melanoma patients clinically assessed by the latest AJCC 8^th^ staging scheme with comprehensive follow-up information for both the discovery and validation stages harmonized at a single institution.

We identified two candidate variants associated with melanoma prognosis (rs60970102 and rs77480547). The alternate allele of these two variants both confer an increased risk of death with a large effect size of HR ~3, consistent with the estimated effect size of previously reported pan-cancer prognostic markers (33). The combined risk alleles (CRA) generated from these two associated variants revealed a significant dosage effect on melanoma OS (HR=2.99 (2.24–3.98), p=9.21×10^-14^). The inclusion of the combined effect of the two discovered variants significantly improved the predictive power for melanoma survival compared to a model with clinical characteristics alone (10-year AUC=76.3 vs AUC=80.7; p=0.007). These two variants highlight MELK and SH3BP4 as putative melanoma prognostic genes, which were further verified by a downstream gene-based analysis. Stratified analyses showed that the effect of the observed associations was largely consistent across different subgroups (age at diagnosis ≥60 vs <60; female vs male; stage I-II vs stage III; tumor of the axial vs extremities), with no evidence of a significant heterogeneity of effect estimates identified in this analysis (p-interaction >0.05).

One of the candidate variants identified in our study was rs60970102. Downstream gene-based analysis revealed that the associations with rs60970102 point to MELK as a candidate gene. MELK (maternal embryonic leucine zipper kinase) is an oncogene in the AMPK serine-threonine kinase family, previously linked with cell cycle regulation, stem-cell renewal, and apoptosis (47). High expression of MELK induces tumor initiation and progression in numerous cancers including melanoma *via* the ATM/CHK2/p53 and NF-kB pathways (48–52). In our study, we observed high MELK expression in both primary and metastatic melanomas (compared to normal tissues), supporting the observations from prior studies and providing additional suggestive evidence that MELK may play a role in both tumor initiation and progression, as also previously reported in an independent study cohort (51). We also noted that high intratumoral MELK expression in primary tumors was associated with worse survival further suggesting its oncogenic function (**Figure 2B**). Similarly, MELKi (inhibitor) treatment of melanoma cells has been shown to block their proliferation and induce cell death (49). To date, there has not been a clinical trial of a MELKi in melanoma; yet, there is an ongoing phase-1 trial of MELKi in advanced breast cancer and triple-negative breast cancer (NCT02926690), suggesting the prognostic and potentially therapeutic relevance of MELK in tumor progression. As such, the prior correlative and causative evidence supporting MELK as a novel therapeutic target in melanoma, along with the suggestive genetic association data from this study, provide further indications in support of a focused investigation of MELK and its role in melanoma progression.

We have also identified rs77480547 as another candidate prognostic marker of melanoma progression. Results from both single-marker analysis of rs77480547 and the downstream gene-based analysis implicate SH3BP4 (SRC homology 3 domain binding protein 4) as a candidate melanoma prognostic marker. SH3BP4 is a gene paralog with approximately 50% shared nucleotide sequences with MACC1 (Metastasis-Associated In Colon Cancer Protein 1), an independent colon cancer prognostic marker (53). Based on The Human Protein Atlas (46), higher expression of SH3BP4 is associated with better survival in stage I-III renal cancer (N=742; log-rank p=8×10^-6^), and the same trend was observed in CM-specific patients, albeit not statistically significant, possibly due to the limited sample size (N=98; log-rank p=0.14; **Figure 2D**). In fact, previous studies have identified SH3BP4 as a tumor suppressor involved in multiple cell growth and proliferation signaling pathways including mTORC1 (54). While there has not been a study to systematically investigate the direct effect of SH3BP4 in the context of melanoma progression, a recent report identified SH3BP4 as a novel pigmentation gene that is inversely regulated by a validated melanoma prognostic marker miR-125b (55–58). The observed effect of miR-125b on melanoma tumor growth has been proposed to be by modulating SH3BP4 expression (55), suggesting the contributing role of this candidate gene in melanoma progression. Pigmentation genes have previously been associated with melanoma risk and prognosis. This, along with our novel finding, suggests a potential link between germline determination of pigmentation and melanoma prognosis. While not an objective of this study, which was focused on white-European ancestries constituting >98% of our study population, we did not have clinical information on pigmentation phenotypes to allow for a more targeted analysis of ancestry/pigmentation effect on prognostic assessment. Additional efforts on this regard should be further examined in more multi-ethnic investigations that would include in-depth clinical and demographic information on pigmentation.

Our study has several major strengths. The study population, consisting of 2 independent cohorts, was ascertained at one cancer center with uniform protocols of surgical treatment of primary tumor removal, uniform collection of harmonized clinical information, and long-term follow-up of primary tumor patients, which both substantially reduce biases of heterogeneous regional and temporal differences in patient care and/or treatment. Also, AJCC 8^th^ staging information was available for every patient for proper adjustment in the analysis, further ensuring that the observed associations were independent of the AJCC staging. Limitations include the lack of information about BRAFi/MEKi targeted therapy, potentially impacting patients’ prognosis, which could be a potential confounding factor in our analysis. In addition, the analysis of other relevant outcomes, such as disease-free or relapse-free survival, not available in this study, would shed more light on potential effects of other systemic therapies. However, given the fact that all patients in this study are ICI-naïve, we suspect that OS, as measured here, would perhaps be the most clinically informative outcome. Also, our analysis is based on a relatively small cohort which may have resulted in an inability to identify other important prognostic markers of smaller effect size. The sample size was also a likely contributing factor for a lack of associations reaching the established GWAS level of significance (p<5×10^-8^). Nevertheless, given a relatively large cohort ascertained at a single institution with harmonized data, and the observed associations with suggestive p<5×10^-7^ in the pooled analysis, it is highly expected that these findings will be replicated externally. As such, a subsequent multi-institutional collaboration is needed to further capture and characterize these signals and additional functional investigations are warranted to elucidate the biological causality of these associations and their impact on melanoma progression.

In summary, in this study, we report the discovery and initial validation of two candidate germline variants associated with CM prognosis. We showed that the inclusion of the combined risk alleles improved the power of melanoma prognostic prediction, as opposed to clinical prognostic variables alone, with a clear dosage effect, further enhancing the possible relevance of the individual associations. Our findings suggest a link between the identified GWAS variants to oncogene and tumor suppressor candidates, with a previously described role in cancer initiation and progression, particularly in CM, as also highlighted by our follow-up transcriptomic and survival analyses. While further functional studies are warranted to expand the biological validity of these findings, the data from this study provide additional suggestive indications that the host germline variation may serve as an independent prognostic factor for better stratification of melanoma patients with a high risk for advanced disease. Pending a large external validation, such personalized prognostic tools will not only enhance more targeted follow-up strategies but may also improve therapeutic guidelines, as immune-checkpoint inhibition (ICI) treatments for melanoma are currently expanding into the neoadjuvant and adjuvant setting. Hence, it will be essential to pool the data from this study with joint ongoing prognostic GWAS efforts from other centers, once published or when the data become publicly available. Such collective initiatives will substantially improve the validation of novel host-related prognostic biomarkers, complementing the current strategies to prevent progression to advanced disease and death in melanoma.

## Data availability statement

The new datasets presented in this article are not readily available due to patient privacy and IRB. Requests to access the datasets should be directed to the authors. Requests to access these datasets should be directed to tomas.kirchhoff@nyulangone.org. The full association results are listed in **Supplementary Table S2** and are available here: https://figshare.com/s/860465237a7250ab82f8. Data from the TCGA is deposited in the dbGaP repository accession number phs000178.v11.p8. Gene expression omnibus (GEO) resources have been used from https://www.ncbi.nlm.nih.gov/geo/ utlizing data under following accession numbers: GSE1239; GSE53462.

## Ethics statement

The studies involving human participants were reviewed and approved by NYU institutional review board (IRB). The patients/participants provided their written informed consent to participate in this study.

## Author contributions

VC, RF, and TK conceptualized and designed the study. VC performed the genotyping experiments. VC, SD, RF, and TK conducted formal data analyses, and/or interpreted the results. VC, RF, and UM harmonized clinical data annotation. VC, RF, TK drafted the manuscript. MS provided genotyping facilities and resources. IO and JW provided patient specimens, clinical data and critical suggestions during manuscript preparation. TK obtained funding and directed the study. All authors contributed to the article and approved the submitted version.
