# Supplementary material for: A genome-wide association study of germline variation and melanoma prognosis
Source: Front Oncol. 2023 Jan 19;12:1050741. doi: 10.3389/fonc.2022.1050741 (PMC9894711; doi:10.3389/fonc.2022.1050741)
Supplement: Supplementary file 1 [file DataSheet_1.docx]

**Supplementary**


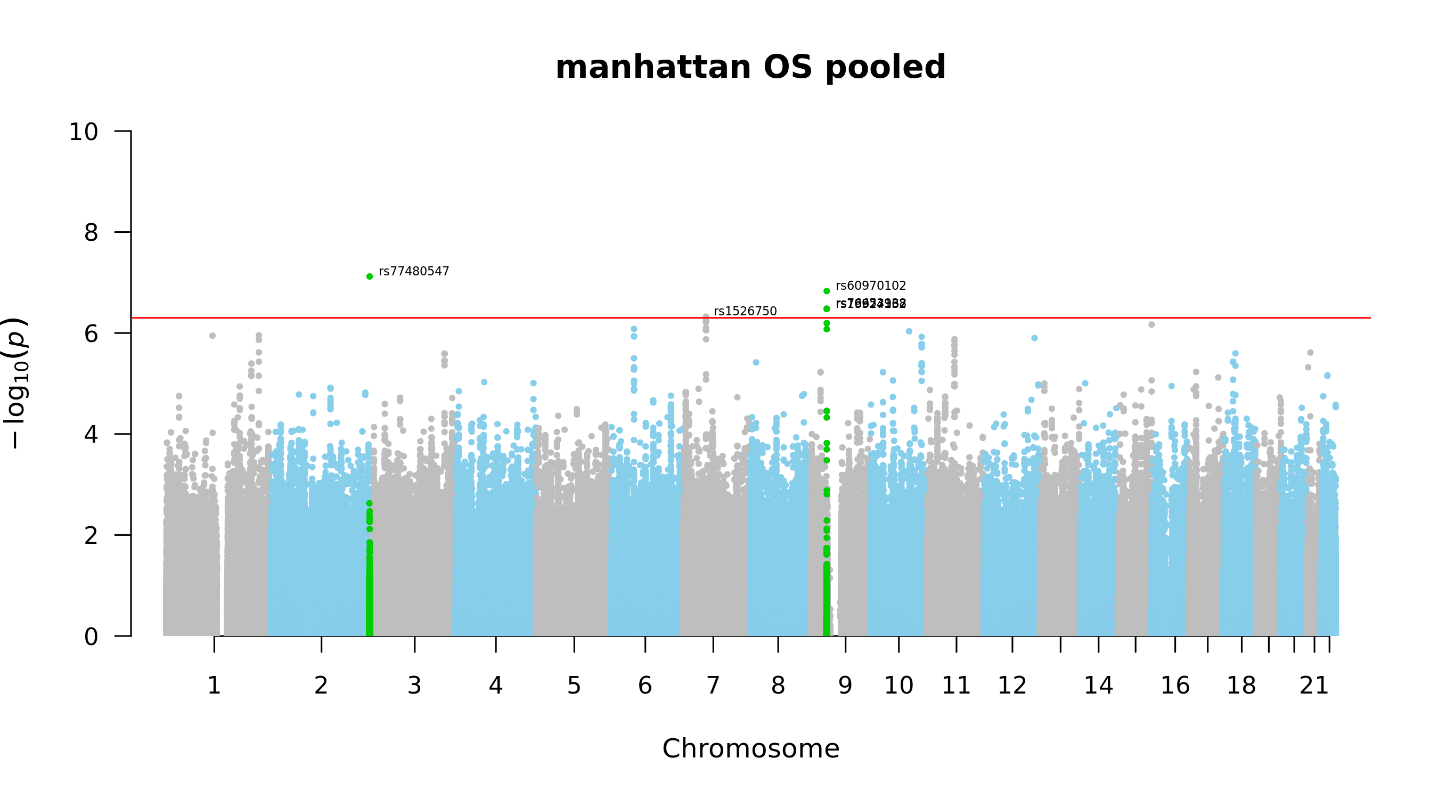


**Figure S1: Manhattan plot of the association between tested germline variants and melanoma overall survival in the pooled analysis**. Wald-test p-values of the associations between germline variants and OS were plotted on the –log_10_ scale. The red horizontal lines indicated GWAS threshold of significance p<5×10^-7^. Three genomic loci were strongly associated with OS (p<5×10^-7^) in the pooled cohort. Only two highlighted loci in green on chromosome 2 and 9 fulfilled the selection criteria (p-value in discovery <10^-4^ with Bayesian false discovery probability BFDP < 0.1; p-value in validation <0.05 with the same directionality of HR with the discovery cohort; and p<5×10^-7^ in the pooled cohort) were reported in the study.

**Table S1: Top associated variants of overall survival validated in the validation phase**. Variants with p <10^-4^ and BFDP< 0.1 in the discovery stage were further investigated in the validation stage (N variants=1,377). Among these, 46 variants were validated in the validation cohort with p < 0.05 and the same directionality of HR with the discovery cohort. We performed SNP clumping with PLINK 1.9 to derive independent index variants (parameters: --clump-kb 250 (genomic distance 250KB) and –clump-r2 0.6 (LD R^2^ > 0.6)). There were 13 clumps formed, in which variants of lowest p-values within each clump were shown in this table. The top 2 variants with boldface and underlined p-values (p <5×10^-7^) were considered statistically significant and reported in the study. Effect estimate HR±95%CI and p-values were computed from multivariable Cox proportional hazard regression, adjusted for age at diagnosis, sex, AJCC stage, tumor anatomic site and top 3 PCs. We further provided genetic annotation of each variant using Haploreg v4 (1)

**B.**

**A.**


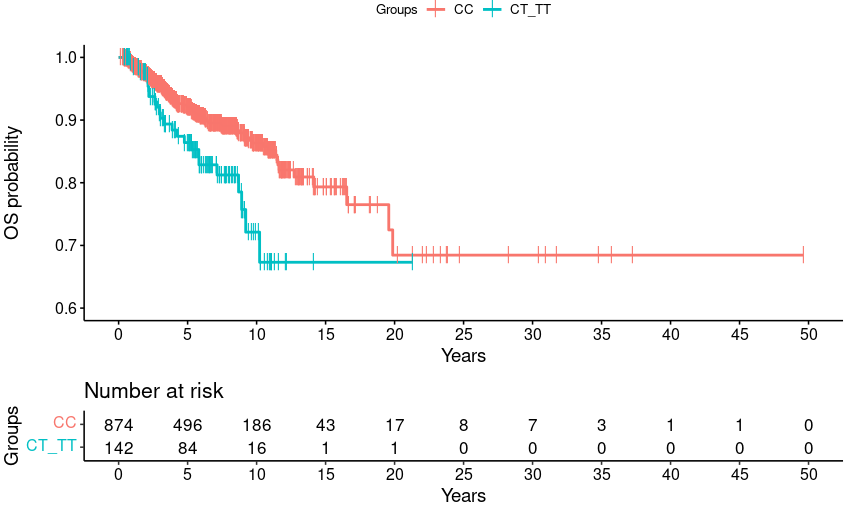

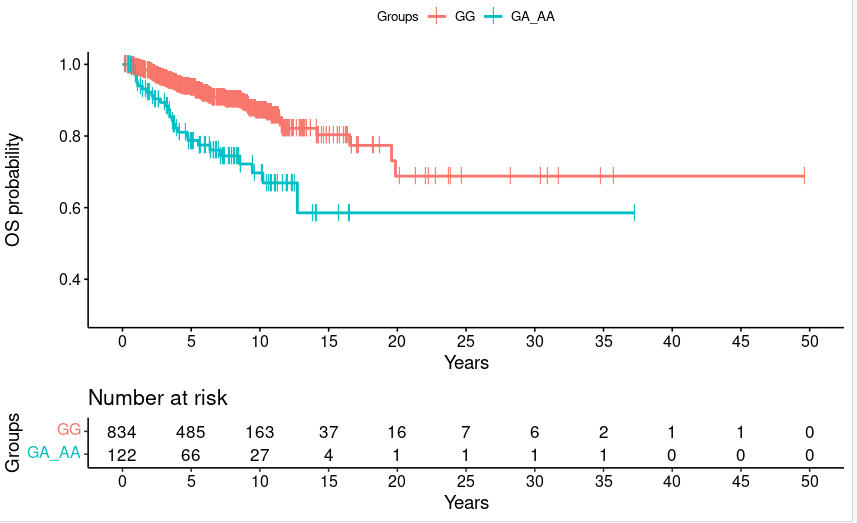


Log-rank p=8E-07

Log-rank p=0.004

**Figure S2: Kaplan-Meire survival curves of the two putative OS markers (rs60970102 and rs77480547 in panel A and B, respectively)**. Due to small number of patients with homozygote alternate genotypes, for better visualization purpose, we combined patients of heterozygote and homozygote alternate genotypes into one group. Log-rank p-values comparing the two survival curves were estimated and shown in the plots.

| **Covariates** | **HR (95% CI)** | **P-value** |
| --- | --- | --- |
| rs60970102 | 3.07(1.97-4.79) | **7.71E-07** |
| rs77480547 | 2.93(1.94-4.41) | **2.72E-07** |
| Age at diagnosis (cont.) | 1.05(1.04-1.07) | 2.37E-11 |
| Stage (cont.) | 2.70(2.10-3.47) | 8.96E-15 |
| Tumor anatomic site (Axial as ref.) | 0.47(0.29-0.75) | 0.001 |
| Sex (Male as reference) | 0.91(0.57-1.45) | 0.70 |
| PC1 (cont.) | 3.82E+03(1.07E-02-1.37E+09) | 0.21 |
| PC2 (cont.) | 7.88E+06(3.17E-05-1.96E+18) | 0.24 |
| PC3 (cont.) | 1.85E-01(3.87E-07-8.84E+04) | 0.80 |

**Table S3: Independent effects of the top two SNPs with melanoma overall survival.** We tested if the two variants (rs60970102 and rs77480547) were independent predictors of melanoma overall survival by fitting both SNPs into a multivariable Cox proportional hazard model, assuming additive genetic effects. Age at diagnosis, AJCC stages, top 3 PC scores were treated as continuous variables (cont.) in the model. We observed independent effects of these two variants as melanoma OS prognostic markers, as evidenced by *Wald-test* p values < 5×10^-7^ in the presence of other covariates.

**Table S4: Estimated 10-year AUC and analysis of variance (anova) comparing model 1 (demographic and clinical predictors alone) vs. model 2 (model 1+ combined genotypes).** Adding the combined germline risk alleles (CRA) into the model significantly improved the AUC prediction (p=0.007) and overall model fit (p=2.4×10^-10^)

| **Models** | **10-year AUC** | **p-value** |  | **Log-likelihood** | **Chisq p-value** |
| --- | --- | --- | --- | --- | --- |
| Age at diagnosis+ Sex + AJCC 8^th^ stages+ Tumor anatomic sites+PC1+PC2+PC3 | 76.3 | Ref. |  | -557.3 | Ref. |
| Age at diagnosis+ Sex + AJCC 8^th^ stages+ Tumor anatomic sites+PC1+PC2+PC3+ **Genotypes** | 80.7 | 0.007 |  | -535.2 | 2.40×10^-10^ |


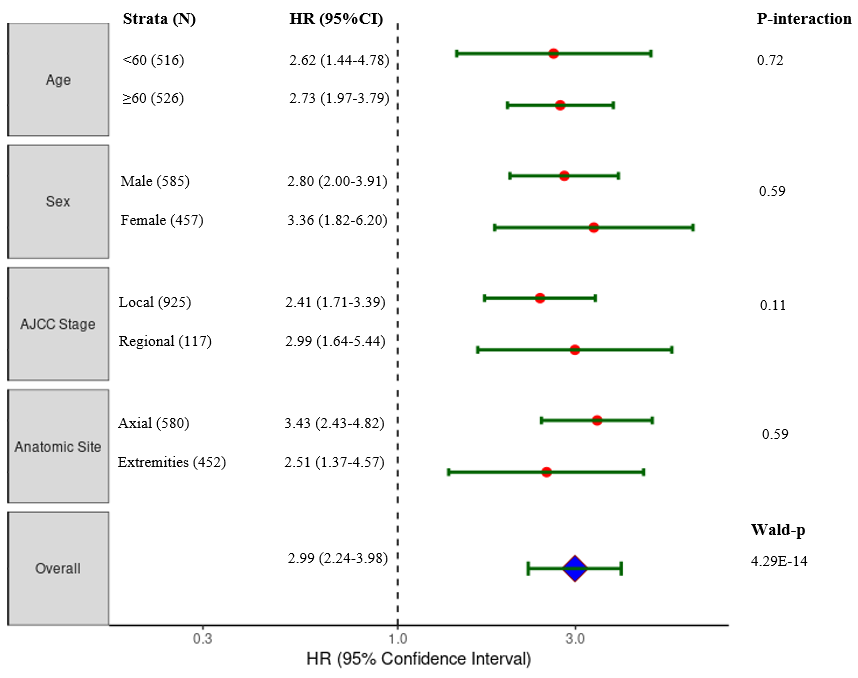


**Figure S3: Stratified analyses of the effect of the composite germline risk score (CRS) on melanoma progression**. Assuming an additive genetic model of CRS, we estimated HR±95%CI using a multivariable Cox-proportional hazard model adjusting for relevant covariates as described in Table S1. We dichotomized covariates as follows: Age of diagnosis (below or above median); Sex (male or female); AJCC stage (local: stage I-II or regional: stage III); tumor anatomic site (Axial or extremities). We observed more pronounced associations between CRG and melanoma progression among younger age, female, tumor stage III and the axial tumor anatomic site. However, p-values for interaction was not statistically significant, suggesting the observed associations were not modified by the tested covariates. The effect estimates in each strata were comparable and consistent with the overall cohort (HR ~3).


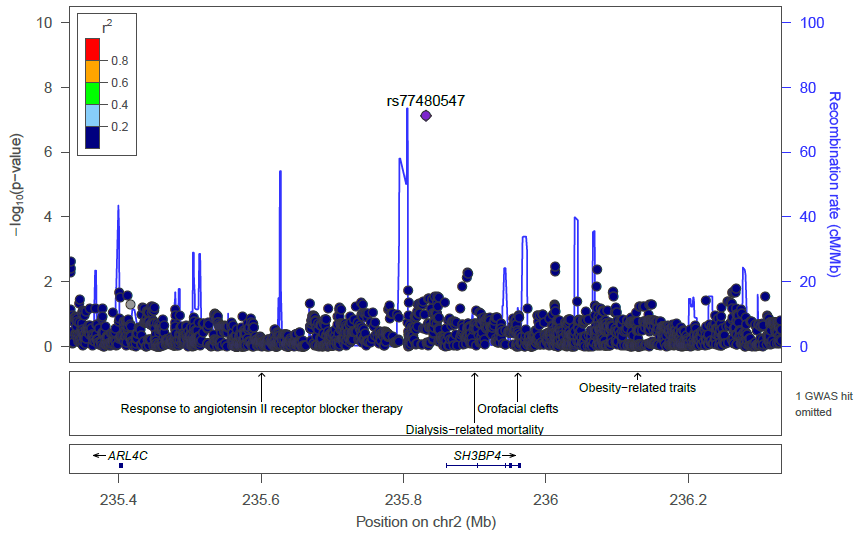

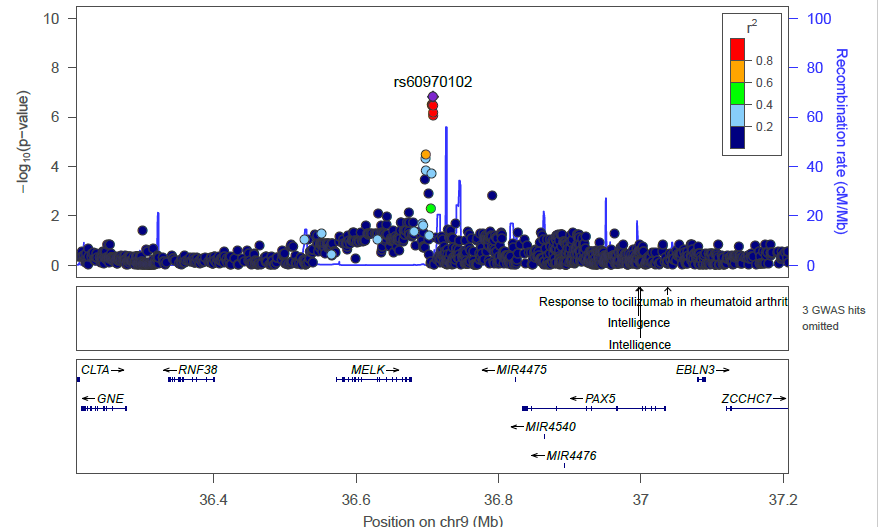


**B.**

**A.**

**Figure S4: Regional plots mapping the top two variants to genes in proximity.** We annotated the top two variants to the genomic regions with ±500KB flank. The closest coding genes for rs60970102 (panel **A**) and rs77480547 (panel **B**) were MELK and SH3BP4 (red arrows pointed), respectively. While rs77480547 had no variant in linkage, there were 5 proxy variants of rs60970102 (R^2^ >0.8 indicated as red dots).

| **Candidate Genes** |  | **Discovery** | |  | **Validation** | |  | **Pooled** | |
| --- | --- | --- | --- | --- | --- | --- | --- | --- | --- |
|  |  | **N SNPs mapped** | **p-value** |  | **N SNPs mapped** | **p-value** |  | **N SNPs mapped** | **p-value** |
| MELK |  | 280 | 1.75×10^-4^ |  | 287 | 0.02 |  | 290 | 1.26×10^-5^ |
| SH3BP4 |  | 483 | 0.007 |  | 493 | 0.009 |  | 488 | 1.13×10^-5^ |

**Table S5: Multi-marker analysis of genomic annotation (MAGMA) of variants mapping within the regions of the two candidate CM prognostic genes from this study: MELK and SH3BP4.** We performed a gene-based association analysis aggregating effects of variants mapped 110KB upstream and 40KB downstream of the candidate genes. Consistent with the implication from a single-variant GWAS analysis, these two genes (MELK and SH3BP4) showed statistically significant associations in both discovery and validation cohorts.

| **Prior candidate variants** | **Pathways** | **Ref.** | **OS-Pvalue*** | **CMSS-Pvalue*** |
| --- | --- | --- | --- | --- |
| rs567403 | Peroxisome | (2) | 0.57 | 0.60 |
| rs7969508 | Peroxisome | (2) | 0.52 | 0.35 |
| rs10889417 | Glycosylation | (3) | 0.98 | 0.39 |
| rs12270446 | Glycosylation | (3) | 0.80 | 0.93 |
| rs9864057 | NADP (Nicotinamide adenine dinucleotide phosphate) | (4) | 0.98 | 0.52 |
| rs12297652 | NADP (Nicotinamide adenine dinucleotide phosphate) | (4) | 0.81 | 0.20 |
| rs11666894 | Endosome-related | (5) | 0.22 | 0.17 |
| rs12376285 | Endosome-related | (5) | 0.92 | 0.96 |
| rs1950902 | Folate metabolic | (6) | 0.14 | 0.08 |
| rs10917006 | Folate metabolic | (6) | NA | NA |
| rs12254548 | Ketone body metabolic | (7) | 0.37 | 0.68 |
| rs71387392 | Ketone body metabolic | (7) | NA | NA |

**Table S6: Previously reported common germline markers of melanoma progression from two-stage candidate gene studies**. We queried our GWAS results of melanoma OS and melanoma-specific survival CMSS to assess if the 12 previously reported CMSS markers show similar associations in our pooled cohort. Ten of the markers were tested in our study using multivariable Cox proportional hazard regression model, while two were removed due to MAF < 5% (indicated as NA). None of the markers showed an association with melanoma outcomes in our cohort. *****Models adjusted for age at diagnosis, sex, AJCC 8^th^ stages, tumor anatomic sites, and top 3 PCs.

**References**

1. Ward LD, Kellis MJNar. HaploReg: a resource for exploring chromatin states, conservation, and regulatory motif alterations within sets of genetically linked variants. Nucleic acids research. 2012;40(D1):D930-D4.

2. Wang H, Liu H, Dai W, Luo S, Amos CI, Lee JE, et al. Association of genetic variants of TMEM135 and PEX5 in the peroxisome pathway with cutaneous melanoma-specific survival. Annals of translational medicine. 2021;9(5).

3. Zhou B, Zhao YC, Liu H, Luo S, Amos CI, Lee JE, et al. Novel Genetic Variants of ALG6 and GALNTL4 of the Glycosylation Pathway Predict Cutaneous Melanoma-Specific Survival. Cancers. 2020;12(2):288.

4. Gu N, Dai W, Liu H, Ge J, Luo S, Cho E, et al. Genetic variants in TKT and DERA in the nicotinamide adenine dinucleotide phosphate pathway predict melanoma survival. European Journal of Cancer. 2020;136:84-94.

5. Lu G, Zhou B, He Y, Liu H, Luo S, Amos CI, et al. Novel genetic variants of PIP5K1C and MVB12B of the endosome-related pathway predict cutaneous melanoma-specific survival. American journal of cancer research. 2020;10(10):3382.

6. Dai W, Liu H, Liu Y, Xu X, Qian D, Luo S, et al. Genetic variants in the folate metabolic pathway genes predict cutaneous melanoma‐specific survival. British Journal of Dermatology. 2020;183(4):719-28.

7. Dai W, Liu H, Chen K, Xu X, Qian D, Luo S, et al. Genetic variants in PDSS1 and SLC16A6 of the ketone body metabolic pathway predict cutaneous melanoma‐specific survival. Molecular carcinogenesis. 2020;59(6):640-50.
